# Quantitative Live Imaging of Endogenous DNA Replication in Mammalian Cells

**DOI:** 10.1371/journal.pone.0045726

**Published:** 2012-09-20

**Authors:** Andrew Burgess, Thierry Lorca, Anna Castro

**Affiliations:** 1 The Kinghorn Cancer Center, Cancer Research Program, Garvan Institute of Medical Research, Sydney, New South Wales, Australia; 2 St. Vincent’s Clinical School, Faculty of Medicine, University of New South Wales, Sydney, New South Wales, Australia; 3 Universités Montpellier 2 et 1, Centre de Recherche de Biochimie Macromoléculaire, CNRS UMR 5237, IFR 122, Montpellier, France; University of Ottawa, Canada

## Abstract

Historically, the analysis of DNA replication in mammalian tissue culture cells has been limited to static time points, and the use of nucleoside analogues to pulse-label replicating DNA. Here we characterize for the first time a novel Chromobody cell line that specifically labels endogenous PCNA. By combining this with high-resolution confocal time-lapse microscopy, and with a simplified analysis workflow, we were able to produce highly detailed, reproducible, quantitative 4D data on endogenous DNA replication. The increased resolution allowed accurate classification and segregation of S phase into early-, mid-, and late-stages based on the unique subcellular localization of endogenous PCNA. Surprisingly, this localization was slightly but significantly different from previous studies, which utilized over-expressed GFP tagged forms of PCNA. Finally, low dose exposure to Hydroxyurea caused the loss of mid- and late-S phase localization patterns of endogenous PCNA, despite cells eventually completing S phase. Taken together, these results indicate that this simplified method can be used to accurately identify and quantify DNA replication under multiple and various experimental conditions.

## Introduction

The replication of genomic DNA must be completed with absolute accuracy, and is one of the most critical steps of cell division. Errors in replication can lead to cell death and or genomic instability, a hallmark of cancer, highlighting its importance. As a result, significant work over several decades has focused on characterizing this critical biological process. However, experiments have primarily been limited to the use of static time-points, which provide only a snapshot of the replication process, thereby limiting our understanding of this biological step. In contrast, the ability to visualize cells in real-time has enabled rapid and numerous advances in our understanding of a wide range of biological processes, such as identifying novel regulators of cell division [1], [2], and uncovering the dynamics of specific protein-protein interactions and modifications [3], [4]. Unfortunately the absence of simple methods for the quantitative live cell imaging of DNA replication has remained a notable stumbling block.

One of the most common visual markers of DNA replication is Proliferating Cell Nuclear Antigen (PCNA). PCNA is a critical component required for the formation of replication factories in the vertebrate nucleus [5]. These factories contain 20–200 replication forks, which together form dozens of globular foci. Consequently, PCNA has been commonly used as an easily identifiable visual signal of active sites of DNA replication [6]–[8]. More recently, over-expressed GFP-tagged versions of PCNA have been used to help visualize DNA replication in live budding yeast [8], drosophila embryos [9] and mammalian cells [6], [7], [10]. While experiments in mammalian cells successfully imaged DNA replication, they were hampered by the need to select cells that were over-expressing only low levels of GFP-PCNA due to the adverse effects that excessive over-expression has on cell cycle progression [6], [11]. However, the unique nuclear localization of GFP-PCNA throughout S phase has allowed DNA replication to be temporally separated into early- (small diffuse foci), mid- (peripheral and nucleoli), and late- (a few very large foci) phases of DNA replication [10]. Unfortunately, there is currently no standardized method that enables the characterization, and classification of significant numbers of cells into these various phases of DNA replication. This combined with the issues associated with the over-expression of GFP-PCNA, has hindered quantitative analysis, and primarily limited the use of previous methods to single cell descriptions of over-expressed PCNA dynamics during DNA replication. Therefore, the development of a simple, robust method for *in cellulo* quantitative analysis of endogenous DNA replication would help advance the field.

Here we report a simplified method that overcomes previous limitations by taking advantage of advancements in modern image acquisition and analysis software and the recent discovery of Chromobodies [12]. Chromobodies are the product of a fluorescent protein (e.g. GFP) fused with a single monomeric antibody fragment isolated most commonly from Camels, Llamas or Sharks. This variable domain (V_H_H or nanobody) is roughly 120 amino acids in length (15 kDa), making it the smallest known intact antigen-binding fragment that can specifically recognize a target protein [13], [14]. One of the first demonstrations of Chromobodies utilized RFP (red fluorescent protein) labeled nanobodies generated against GFP (green fluorescent protein). Cells expressing the RFP Chromobody were then transfected with GFP fused either H2B, ß-Actin or PCNA to verify that nanobodies could successfully label various subcellular structures [12]. This result combined with their extremely small size, high stability, and excellent reproducibility, suggests that Chromobodies are perfectly suited to live cell imaging and quantification and tracking of endogenous proteins [12], [15], [16]. In this study we took advantage of a new, but untested clonal cell line, which stably expresses a Chromobody that specifically targets endogenous PCNA. By taking advantage of their consistent cell-to-cell expression levels we were able perform quantitative analysis across multiple replicates, and experimental conditions, providing detailed real time high-resolution data of endogenous replication in cells.

**Figure 1:**
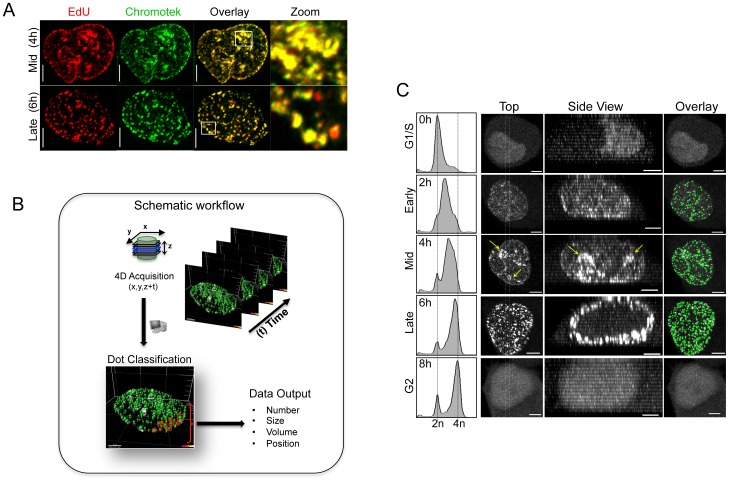
Using a novel Chromobody cell line to image DNA replication in live cells. (**A**) HeLa Chromobody (Chromotek) cells were blocked in G1/S with thymidine for 24 h. Cells were released and then pulsed for 20 min with 10 µM EdU (Invitrogen) at 4 h and 6 h post release to label mid- and late- replicating cells. Cells were fixed and stained using the EdU Click-iT labeling kit as per the manufactures instructions (Invitrogen). Shown are the maximum image projections from 0.3 µm serial Z-sections. High levels of co-localization (Yellow) are clearly observed in the overlaid images between the PCNA targeting Chromobody (Green) and EdU incorporation (Red), Scale bare equals 5 µm. (**B**) Schematic workflow showing the simplified method used in this study to capture, process and analyze data. (**C**) HeLa Chromobody cells (Chromotek), were synchronized in G1/S and analyzed by 4D microscopy (3D + time). Duplicate samples were taken every 2 h and processed by flow cytometry to confirm that the majority of cells were progressing through S phase. Shown are the maximum projection images (Top) from a typical cell as it progresses through S phase. Dotted lines indicate the position of the Z-section slice taken for the side- view (Side) yellow arrows indicate the nucleoli. The results from the automated dot-tracking feature from Imaris software (Bitplane Inc.) are also shown (Overlay). Scale bar 5 µm.

## Materials and Methods

### Cell Culture and Synchrony

Experiments were performed on the HeLa Chromobody cell line (Chromotek), which stably expresses a PCNA targeting Chromobody fused to GFP [12]. Cells were seeded onto a 35 mm glass bottom culture dish, and blocked in G1/S for 24 h in 2.5 mM Thymidine. Cells were then washed 3 times with pre-warmed media, and released from G1/S by adding fresh media supplemented with 25 µM 2′-Deoxycytidine. Cells were immediately placed on the microscope for live imaging. In some cases cells were treated with low dose Hydroxyurea (200 µM) at the point of release from thymidine.

### Flow Cytometry

Flow cytometry analysis (FACS) was performed as previously described [17]. Briefly, cells were fixed in ice-cold 70% ethanol and stored at −20°C. Samples were then washed once in phosphate-buffered saline and re-suspended in DNA staining solution consisting of 5 µg/ml propidium iodide and RNase A (0.5 mg/ml) in phosphate-buffered saline. Cells were analyzed on a FACS Calibur (BD Biosciences) using Cell Quest Pro software.

**Figure 2:**
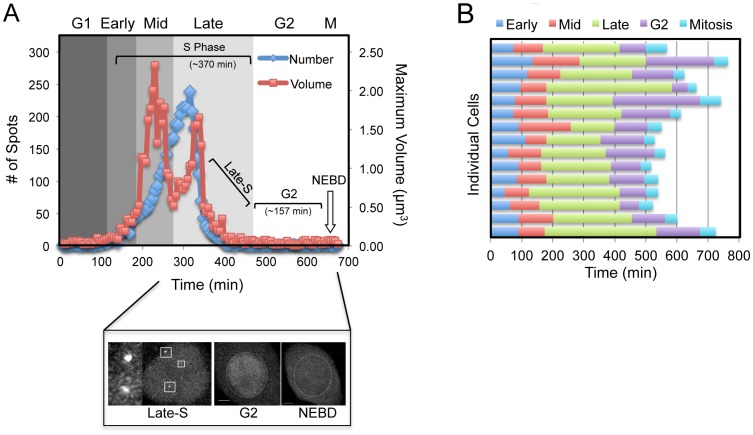
Quantification of Live Imaging Data. (**A**) Dot quantification data was exported from Imaris, and compiled into graphs. The total number of dots was plotted and overlaid with the maximum foci volume at each time point. Cells were separated into G1, early-, mid-, late- S phase, G2 and Mitosis (M) as per methods, shown is a representative graph from a single cell over time. Inset shows typical maximum projection images of late- S, G2 and early mitotic cells. Dotted white line indicates location of the nuclear envelope before and after breakdown (NEBD). (**B**) A compilation bar graph detailing the high level of reproducibility in timing of each cell cycle phase (G1, early-, mid-, late-, G2, Mitosis) for 15 individual control cells.

### Live Imaging

Images were acquired using a Zeiss LSM780 inverted confocal laser scanning microscope fitted with a Zeiss Plan-Apochromat 63x/1.4 oil DIC lens. Images were acquired with a 1AU pinhole size, with 21 Z-section at 0.75 µm steps, taken every 5 minutes for up to 16 h. A single excitation laser wavelength of 488 nm (490–551 nm filter) at a minimal 0.200% laser intensity, and short 3.15 µsec pixel dwell, was used to minimize cell toxicity. Images were recorded at a frame size of 1028×1028 pixels, a pixel size of 132 nm, and zoom of 1.0x. Cells were maintained in a heated 37°C chamber and stage, with 5% CO2, to ensure optimal growth conditions throughout the time-lapse.

**Figure 3:**
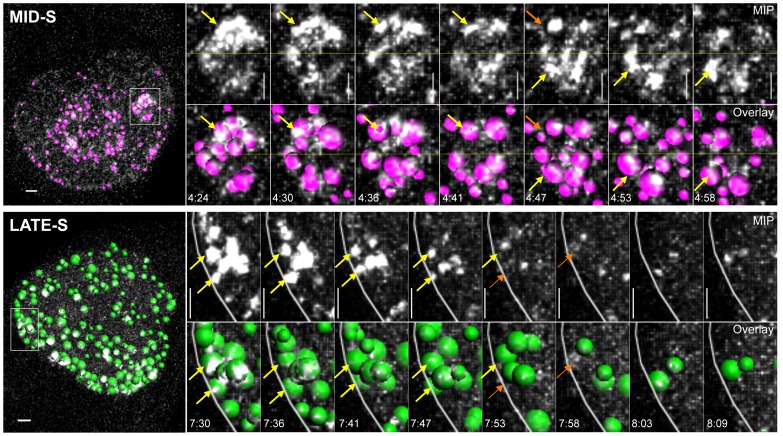
Individual dots resolve before moving to other replication sites. Maximum image projection (MIP), and automated dot tracking overlay (Overlay) images from a mid- (purple) and late- S (green) phase cell, and the corresponding zoom cropped sections highlighting the nucleoli and peripheral foci respectively. Horizontal segregation of the nucleolus (yellow dotted line) clearly shows the top half undergoing replication at earlier time points, before shifting to the lower half. However, over time no significant movement of individual dots was observed in either mid- or late- locations (yellow arrows), orange arrows indicates point where a focus is resolved. Scale bar 1 µm, time expressed as hh:mm.

### EdU Labeling and Immunofluorescent Staining

HeLa Chromobody cells were grown on Histogrip (Invitrogen) coated glass coverslips. Cells were synchronized and pulsed with 10 µM EdU for 20 minutes at 4 and 6 hours post release to label mid-, and late- S phase cells respectively. Cells were then fixed for 10 minutes in PBS with 3.7% formaldehyde and 0.5% Triton X-100. All cells were washed and blocked (3% BSA, 0,1% tween 20 in PBS) for 30 min. Click-iT- EdU Alexa 555 staining was performed as per manufacturers instructions (Invitrogen). Coverslips were mounted using Prolong Gold mounting medium (Invitrogen) and captured using a Leica DM6000 microscope coupled with a Coolsnap HQ2 camera, using a Leica 100X APO 1.4 lens, powered by Metamorph 7.1 (Molecular Devices) software. Serial 0.3 µm Z-sections were taken, and deconvolved using Huygens 3.0 software (Scientific Volume Imaging). Maximum projections were performed with Image J, false coloring and overlays were performed using Photoshop CS5 Extended software (Adobe).

**Figure 4:**
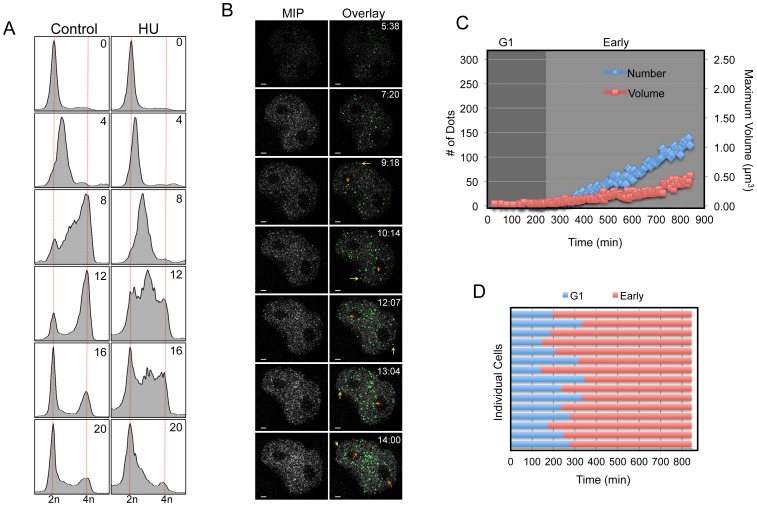
Low dose HU blocks mid and late origins of replication. (**A**) DNA content FACS analysis of thymidine synchronized HeLa Chromobody cells treated with 200 µM of HU at time of release. Cells were harvested at 0, 8, 12, 16, and 20 hours after release from G1/S. Data clearly shows that HU treated cells eventually complete S phase and transit through mitosis and back into G1 phase after 16–20 hours. (**B**) Shown are the maximum image projections (MIP) and the corresponding overlaid Imaris dot classification in green (Overlay) from time-lapse movies of HeLa Chromobody cells synchronized and treated with low dose Hydroxyurea (200 µm). Time is expressed in hh:mm, scale bar is 2 µm. Arrows highlight examples of nucleoli (orange) and peripheral (yellow) foci localizations. (**C**) As per Figure 2A, except as cells were released from G1/S they were treated with low dose Hydroxyurea (200 µM). Shown is a representative graph from a single cell over time. (**D**) A compilation bar graph detailing the fate of 15 individual HU treated cells.

### Image Analysis

4D (3D+time) data sets were imported into Imaris v7.32 (Bitplane) software. Cells were then individually isolated in time and space, using the Spot-tracking feature of Imaris. Individual spots were defined with a variable and initial size estimate of 0.5 µm. A stringent quality threshold was placed over the image to minimize any background spots appearing outside of the nucleus. Using this method, greater than 99% of spots were accurately identified, with any errant dots removed manually before exportation of raw data to Excel (Microsoft). Numerous characteristics, including the three-dimensional volume, number of spots and their intensity at each time-point were recorded, exported to Excel, and compiled into graphs. In some cases, data was exported into GraphPad Prism 5 (La Jolla, CA) to create box-plots and perform statistical analysis. Although mean volume, diameter and maximum volume followed a similar pattern (Supplementary Figure S1C), maximum volume provided a greater overall change, which helped provide clearer segregation between early-, mid- and late- S phase. Control graphs were then annotated and cross-referenced with the raw image data to accurately classify early-, mid-, late-, G2, NEVB (nuclear envelope breakdown), and mitotic exit. Early- S phase was characterized by a steady increase in both number and volume of foci over the background reading. As cells entered mid- S phase, there was a dramatic increase in dot volume, reaching its peak in mid- S phase. The shift from the central nucleolar staining to the periphery of the nucleus, corresponded with a sharp drop and then rise in focus volume, along with the peak in the total number of foci. Completion of late- S phase and entry into G2 phase was scored as the resolution of the last remaining focus. Entry into mitosis was easily observed by NEVB, which visually corresponded with a rapid diffusion of GFP staining from the nucleus to the cytoplasm and rounding of the cell. Imaris was also used to export 2D maximum image projection images and movies, with images optimized and compile into montages using Photoshop CS5 Extended (Adobe).

## Results

### Characterization of the Novel HeLa Chromobody Cell Line to Quantify DNA Replication in Real Time

In this study, we took advantage of the recent development of a novel stably expressing HeLa Chromobody cell line, to accurately follow the cell cycle dynamics of endogenous PCNA in real time. Chromobodies have previously been used to successfully track exogenous GFP fusions and endogenous cellular proteins such as lamin B1 [12]. However this is the first publication utilizing a Chromobody designed to specifically target endogenous PCNA. Significant co-localization between sites of EdU incorporation and GFP signal confirmed the specificity of the PCNA Chromobody to accurately identify sites of active DNA replication (Figure 1A). To analyze DNA replication *in cellulo*, we employed a highly simplified workflow, to help maximize ease of use and throughput (Figure 1B). Briefly, HeLa Chromobody cells were synchronized in G1/S by thymidine block, released and imaged every 5 minutes for 16 hours. The raw image data was imported into Imaris software (Bitplane) and reconstructed in 4D (3D + time) data sets. Next, individual cells were analyzed using the semi-automated dot-tracking feature of Imaris, with the number, size (volume, diameter), intensity and position recorded (Movie S1). Using the previously reported changes in localization of over-expressed GFP-PCNA as a guide [6], [7], [10], entry into early- S phase was classified based on the shift from the homogenous diffuse nuclear Chromobody signal, characteristic of G1, to a speckled appearance with dozens of small ‘dots’ throughout the nucleus, indicating that endogenous PCNA had become associated with the leading strands of active origins of replication (Figure 1C, Movie S2). As cells entered mid- S phase the number of foci increased and their size dramatically enlarged, correlating with a shift of staining from diffuse nuclear dots to primarily around the nucleoli. As cells entered late- S phase, the localization shifted from the interior and nucleoli, to the periphery of the nucleus. We were able to clearly distinguish these two localizations both temporally and visually in 3-dimensional space (see side view Figure 1C), with the peripheral localization occurring primarily after nucleolar localization (Figure 1C). The nucleoli and peripheral localizations could be separated graphically by plotting the number and maximum volume of foci over time. The first peak in focus volume corresponded with nucleolar staining, while the shift from nucleoli to the periphery resulted in a rapid drop followed by a second smaller peak in focus size. During this second peak in volume, the total number of dots reached its maximum, after which both parameters rapidly declined (Figure 2A, and Supplementary Figure S1). Across the 15 cells analyzed there was a high level of reproducibility, with the mean and standard error of each phase length being 86 +/− 6 minutes, 102 +/− 7 minutes, and 244 +/− 17 minutes for early-, mid- and late- S phase respectively, with total S phase length averaging 7.2 hours (Figure 2B, and Supplementary Figure S2). This correlated with flow cytometry DNA content analysis, which showed that the peak of synchronized cells completed S phase within 6–8 hours (Figure 1C). In addition, accurate estimation of G2 length (129 +/− 15 minutes) was also possible by calculating the time between the resolution of the last PCNA focus and NEVB (Figure 2A, 2B, and Supplementary Figure S2). In agreement with previous studies [7], we did not observe nor were able to track significant movement of individual dots within the microenvironment (Figure 3, and Supplementary Figure S3). Furthermore, individual focus intensity generally decreased over time, and we were not able to track any focus moving from one location to another. This was most obvious at the nucleoli where individual dots in the upper section of could be seen to resolve, while new sites was born on the lower side (Figure 3, mid-S).

### Low Dose Hydroxyurea Inhibits Mid and Late S Phase Foci

To test the ability of this method to identify, quantify and characterize defects in DNA replication, we treated G1/S synchronized cells with a low concentration (200 µM) of Hydroxyurea (HU) as they entered S phase. At this concentration, HU activates the Chk1 DNA damage pathway, which leads to a reduction in the number of active replication factories by preventing the firing of late origins [11], [18]–[20]. Therefore, HU treatment should result in a reduction in both the number and volume of PCNA foci, and an increase in time taken to complete S phase. This is what we observed in our system, with the majority of HU treated cells taking at least twice as long (16–20 hours) to complete S phase, compared to ∼8 h for controls (Figure 4A and Supplementary Figure S2). Visually the phenotype of HU treated cells resembled early- S phase throughout the ∼15 h time frame, with numerous diffuse small foci. Although individual foci could be randomly observed throughout all sub-nuclear localizations, there was no obvious accumulation at the nucleoli or at the periphery of the nucleus at any specific time point (Figure 4B, and Movie S3). In agreement, quantification showed that HU treated cells failed reach a significant peak in both the number and volume of foci during the time course (Figure 4C, Movie S4), which was highly reproducible (Figure 4D, and Supplementary Figure S4A). The number of dots slowly increased to a median average of only 65, compared to 282 in controls (Supplementary Figure S4B). Similarly, the average maximum dot volume reached only 0.34 µm^3^ for HU treated cells, which was more than 7-fold less than the 2.66 µm^3^ reached in controls. Likewise, there was a highly significant (p<0.0001) reduction in the total number of PCNA foci over the 16-hour timeframe with the average median number of foci only 2351 in HU cells compared to 6125 in controls (Supplementary Figure S4C).

## Discussion

In this study we utilized and characterized for the first time a new Chromobody cell line that specifically labels endogenous PCNA. The Chromobody cell line accurately labels sites of active DNA replication, and showed a high level of consistency in phenotypes across both controls and HU treated samples, enabling the quantitative modeling S phase under both normal and stressed conditions.

In control cells, we observed a highly ordered and synchronous progression from early-, to mid-, and then late- S phase, which resulted in highly coordinated PCNA sub-cellular localizations for each of these states (diffuse foci, nucleoli, and the periphery of the nucleus respectively). In contrast, treatment with low dose Hydroxyurea more than doubled S phase transit time, and completely disrupted the normal pattern of PCNA localization. Specifically, HU treated cells failed to show the synchronous pattern of replication seen during mid- (nucleoli) and late- (periphery) S phase. Although small foci could be observed randomly throughout the time-course at these localizations, indicating that cells were able to eventually replicate these areas of the genome. These results correlate with previous reports that utilized static time points of cells pulse labeled with Cy3-dUTP and over-expression of GFP-PCNA, which showed that the activation of late origins of replication are inhibited, and fork speed is dramatically reduced by low dose treatment with HU [11], [18]–[20], [25]. This resulted in a redirection of replication away from completely unreplicated regions and towards active factories [11], which prevented replication forks from stalling and enabled cells to slowly but eventually complete DNA replication.

Surprisingly, in control cells the sub-cellular localization of the Chromobody was similar, yet subtly different to that of previous studies, which utilized over-expression of GFP-PCNA [6], [7], [10]. Specifically, unlike previous studies that reported both nucleolar and peripheral staining during mid-S phase, we clearly distinguish these two localizations temporally, with peripheral staining occurring primarily after nucleolar staining. This maybe due to the improved confocal imaging system used in this study, which allowed us to image both time and space in higher resolution than previous studies. Secondly, we did not observe the large inter-nuclear (>1 µm diameter) globular structures that previous GFP-PCNA studies characterized as late-S phase [6], [7], [10]. Again its possible that the higher resolution images obtained in this study allowed us to visually separate two closely localized dots that may have previously been classified as a large singular focus. In support, super-resolution 3D-SIM microscopy analysis of static images of cells over-expressing GFP-PCNA showed a three to five fold increase in the total number of foci compared to conventional light microscopy [21], suggesting perhaps that previous studies may not have been able to distinguish between two or more closely localized dots. However, we have previously observed that excessive over-expression of some GFP-tagged proteins can result in the accumulation of large globular nuclear foci [22], which are often associated with sites of protein degradation [23]. Therefore, it is possible that some of these large, intra-nuclear late-S phase foci could be due to cells actively trying to remove excess levels of GFP-PCNA, rather than marking sites of late replication.

In agreement with previous exogenous studies [24], we were not able to observe significant movement of an individual focus within the nuclear microenvironment. At the magnification level used in this study, we were only able to resolve at the level of the replication factory, which can contain from 20–200 individual replication forks. At this resolution it is not possible to track the movement of individual PCNA rings along a single DNA replication fork. Thus, at the level of replication factories, endogenous PCNA appears to behave similarly to the exogenous GPF-tagged protein, dissociating from early factory sites into the nucleoplasmic pool before re-associating with later sites of replication.

In summary, our data suggests that the pattern of endogenous DNA replication is defined by the presence of small diffuse foci during early- S phase, followed by replication of nucleolar regions during mid- S phase, with late- S phase marked by the replication of sites at the periphery of the nucleus, which are greater in number but smaller in size than those at nucleolar sites during mid- S phase. Taken together, these results highlight the subtle yet important effects that improved imaging and the over-expression of exogenous proteins can have on cell biology, and emphasizes the advantages of being able to image endogenous proteins with Chromobodies.

In conclusion, we present here a greatly simplified and highly reproducible method for visualizing, quantifying, and segregating cells into early-, mid-, and late- phases of DNA replication *in cellulo.* This method is robust enough to accurately model endogenous DNA replication across a population of cells, and avoids the many issues associated with previous protein over-expression methods. The use of the novel Chromobody targeting endogenous PCNA also enabled excellent temporal resolution allowing for new insights into the location and classification of early-, mid-, and late- stages of DNA replication. Furthermore, this system allows additional variables such as drug manipulations to be used, and could be adapted for siRNA knockdown, over-expression, or high content screening. Finally, it benefits from the use of a homogenous stable expressing Chromobody cell line, and off the shelf equipment and software, making it highly cost effective, simple and accessible to a broad cross-section of the research community.

## Supporting Information

Figure S1 (**A**) Additional graphs for control cells as shown in Figure 2A. (**B**) Shown is a bar graph plotting the volume (µm^3^) and diameter of all of the individual dots counted for a single control cell. The general pattern of the maximum volume closely mirrored that of the average volume and diameter.
(TIF)

Figure S2 Box plot with mean and 5–95% confidence intervals comparing the total time taken to complete each cell cycle phase across 15 control and 15 HU treated cells.
(TIF)

Figure S3 As per Figure 3, an additional images from a Late-S phase cell showing the maximum projection (MIP), and automated dot tracking overlay (Overlay) in purple.
(TIF)

Figure S4 (**A**) Additional graphs for HU treated cells as shown in Fig. 4C. (**B**) The maximum number of dots and maximum volume reached from Control and Hydroxyurea treated cells (HU 200 µM), is presented as Box plots with 5 to 95% confidence intervals. (**C**) A Box plot of the mean and 5–95% confidence intervals comparing the total number of dots counted across 15 control and 15 HU treated cells. Two-tailed unpaired Student-t tests were performed to determine statistical relevance; significant P values (***) are shown (p-value <0.0001).
(TIF)

Movie S1 Time-lapse 3D render of a typical field of control HeLa Chromobody cells overlaid with the dot-tracking results produced from Imaris software.
(MP4)

Movie S2 Video montage of a typical control HeLa Chromobody cell progressing from G1, through S phase, G2 and then undergoing mitosis. Shown are the 2D (x–y) maximum projected images and a 2 µm ortho-slice (y–z), with and without dot tracking.
(MP4)

Movie S3 Time-lapse 3D render of a typical field of Hydroxyurea (200 µM) treated HeLa Chromobody cells overlaid with the dot-tracking results produced from Imaris software.
(MP4)

Movie S4 Video montage of a typical Hydroxyurea (200 µM) treated HeLa Chromobody cell. Shown are the 2D (x–y) maximum projected, and 2 µm ortho-slice (y–z) images, with and without dot tracking.
(MP4)
