# Supplementary figures and images for: Quantitative Live Imaging of Endogenous DNA Replication in Mammalian Cells
Source: PLoS One. 2012 Sep 20;7(9):e45726. doi: 10.1371/journal.pone.0045726 (PMC3447815; doi:10.1371/journal.pone.0045726)

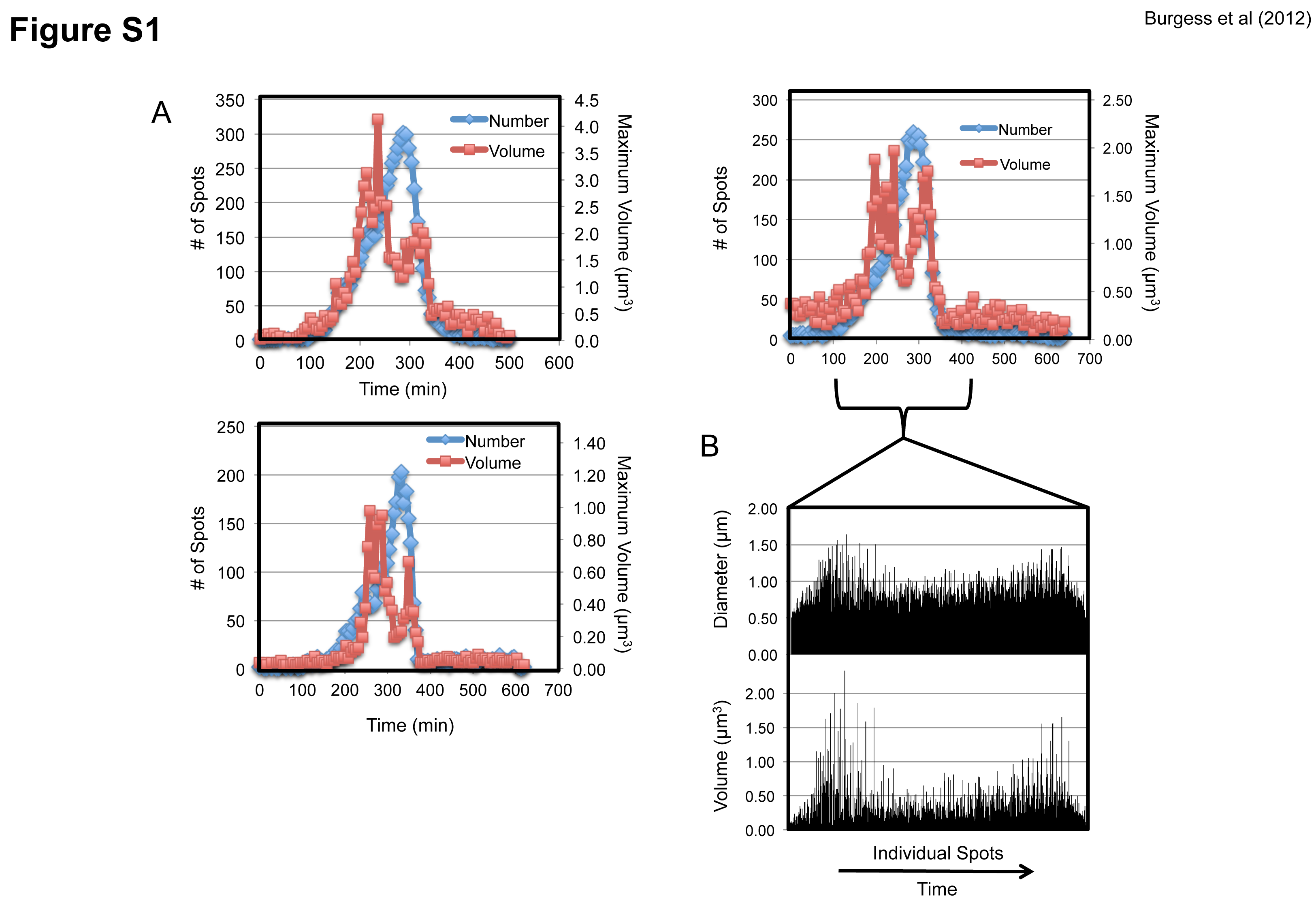

Supplement: Figure S1 — (A) Additional graphs for control cells as shown in Figure 2A. (B) Shown is a bar graph plotting the volume (µm3) and diameter of all of the individual dots counted for a single control cell. The general pattern of the maximum volume closely mirrored that of the average volume and diameter. (TIF) [file pone.0045726.s001.tif]

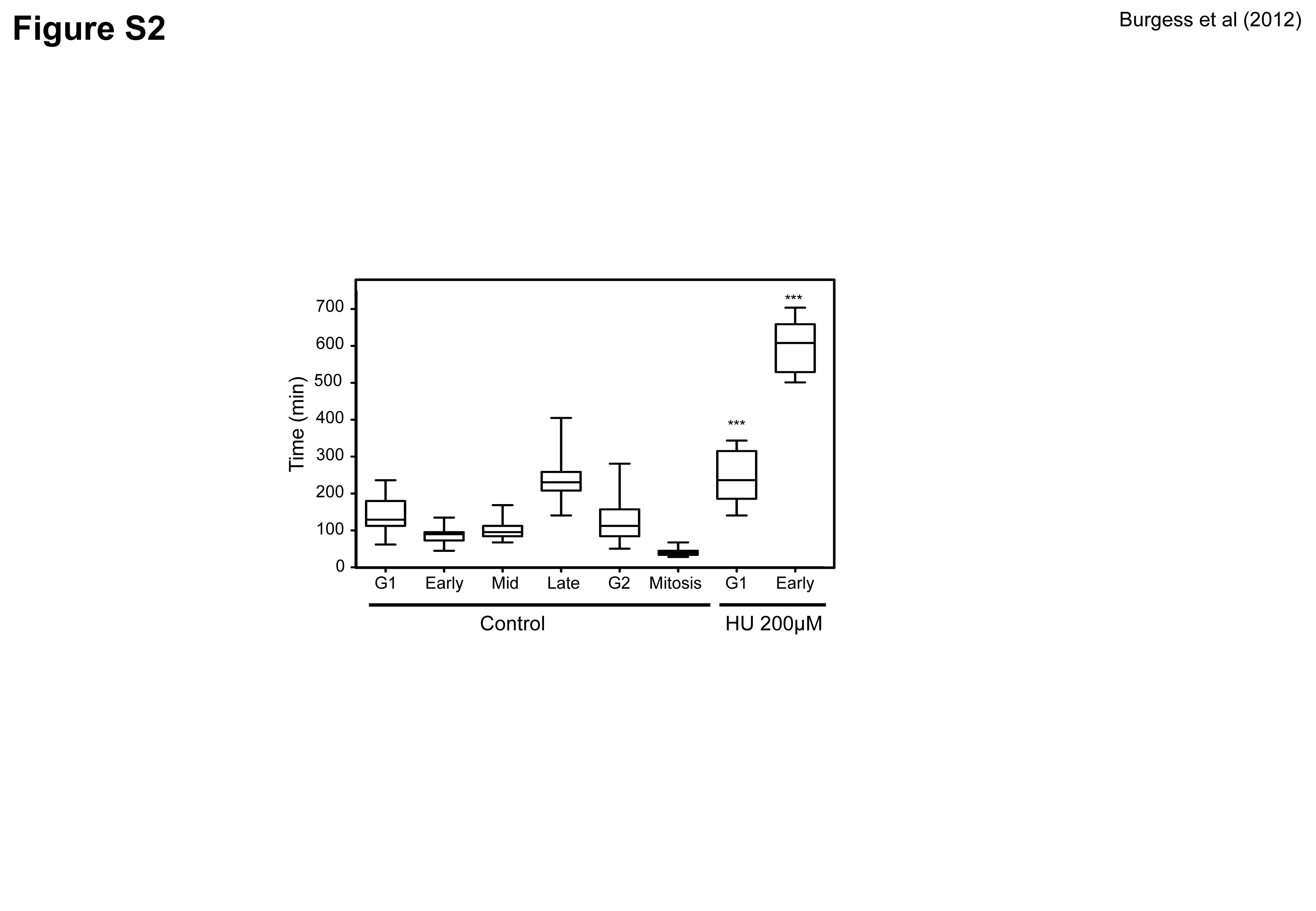

Supplement: Figure S2 — Box plot with mean and 5–95% confidence intervals comparing the total time taken to complete each cell cycle phase across 15 control and 15 HU treated cells. (TIF) [file pone.0045726.s002.tif]

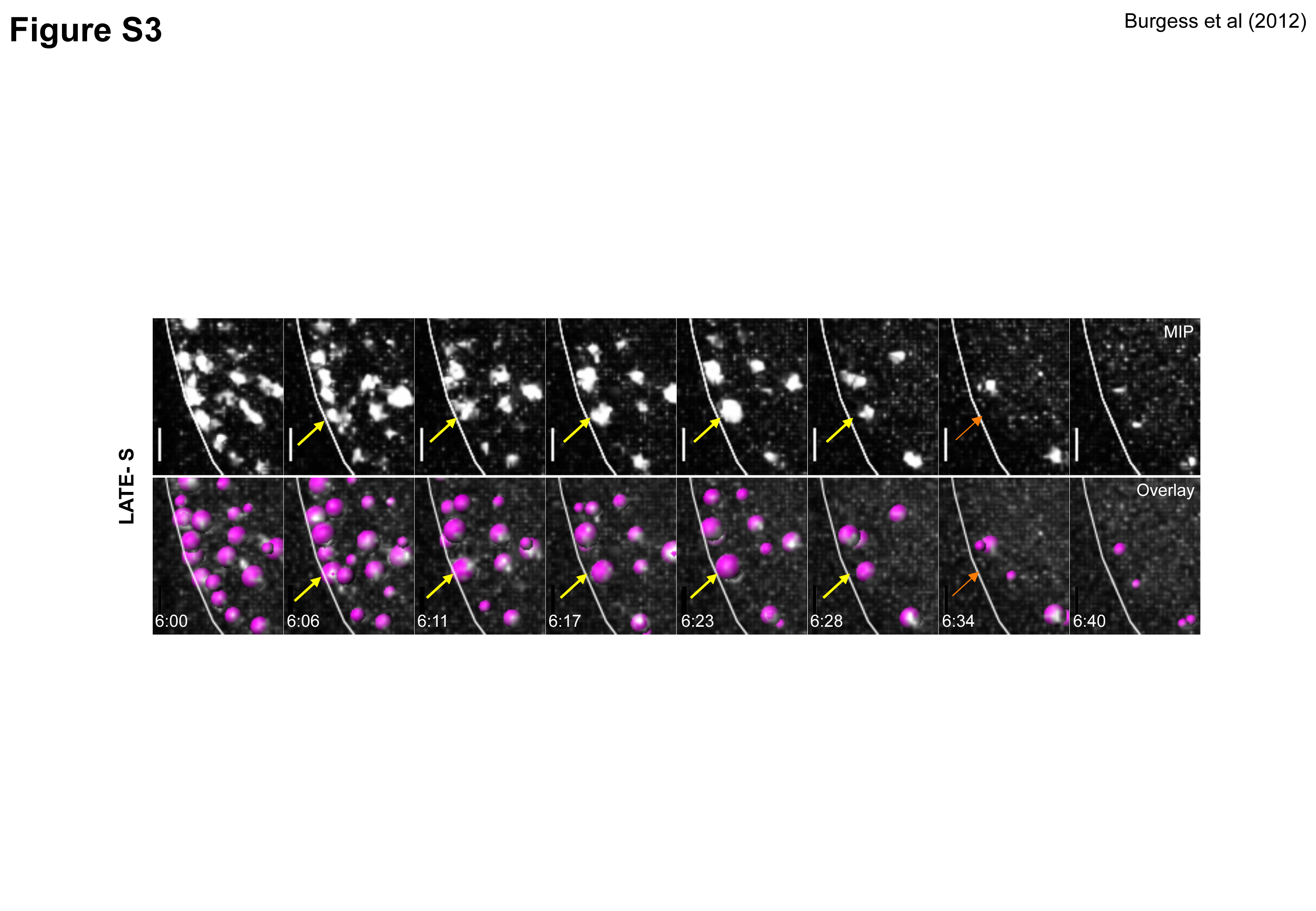

Supplement: Figure S3 — As per Figure 3, an additional images from a Late-S phase cell showing the maximum projection (MIP), and automated dot tracking overlay (Overlay) in purple. (TIF) [file pone.0045726.s003.tif]

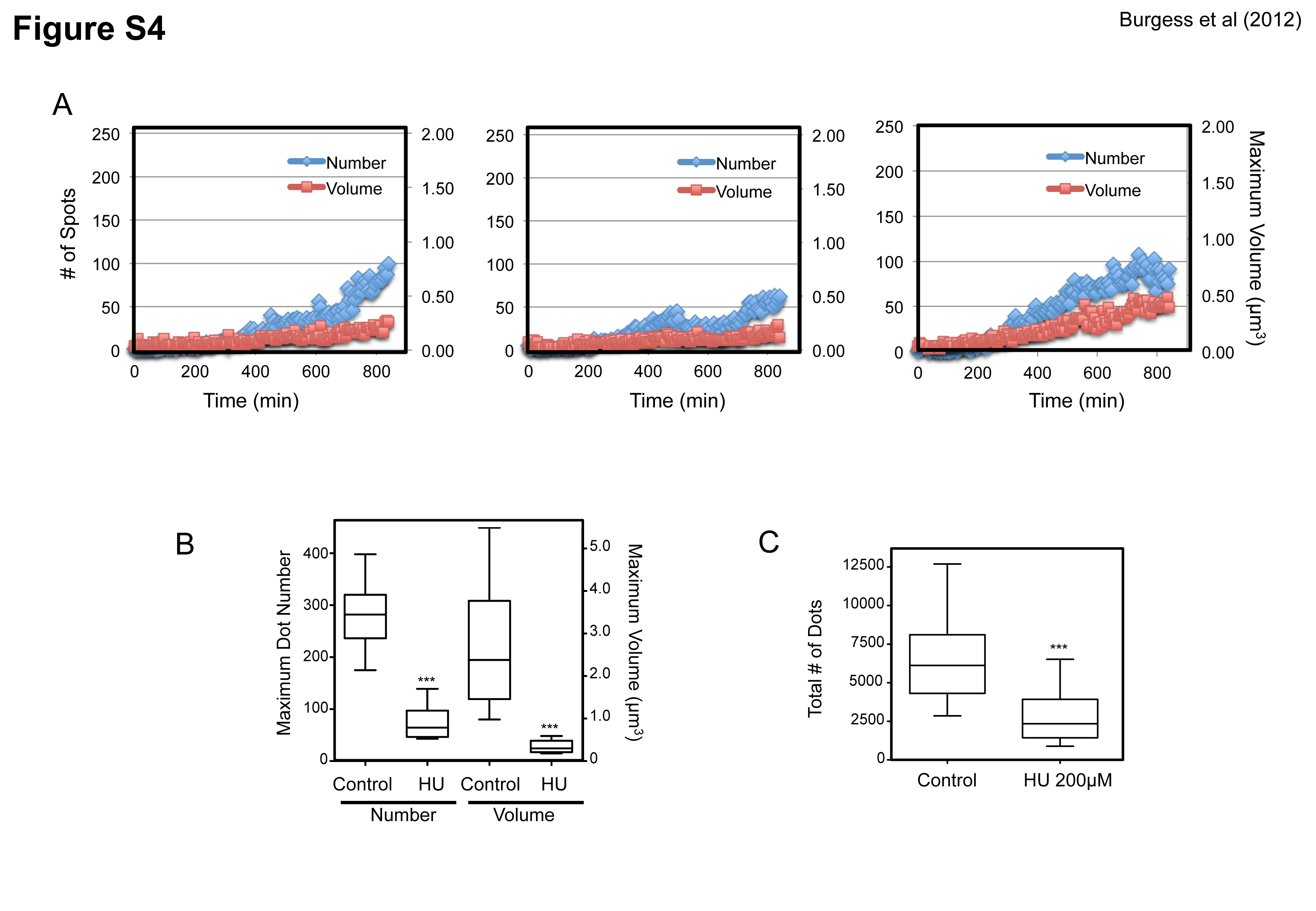

Supplement: Figure S4 — (A) Additional graphs for HU treated cells as shown in Fig. 4C. (B) The maximum number of dots and maximum volume reached from Control and Hydroxyurea treated cells (HU 200 µM), is presented as Box plots with 5 to 95% confidence intervals. (C) A Box plot of the mean and 5–95% confidence intervals comparing the total number of dots counted across 15 control and 15 HU treated cells. Two-tailed unpaired Student-t tests were performed to determine statistical relevance; significant P values (***) are shown (p-value <0.0001). (TIF) [file pone.0045726.s004.tif]
